# The Relation between Eating Habits and Abdominal Fat, Anthropometry, PON1 and IL-6 Levels in Patients with Multiple Sclerosis

**DOI:** 10.3390/nu12030744

**Published:** 2020-03-11

**Authors:** Eraci Drehmer, Jose Luis Platero, Sandra Carrera-Juliá, Mari Luz Moreno, Asta Tvarijonaviciute, Marí Ángeles Navarro, María Mar López-Rodríguez, Jose Enrique de la Rubia Ortí

**Affiliations:** 1 Department of Basic Sciences, Catholic University of Valencia San Vicente Mártir, 46900 Torrent, Spain; eraci.drehmer@ucv.es (E.D.); ml.moreno@ucv.es (M.L.M.); angeles.navarro@ucv.es (M.Á.N.); 2 Doctoral Degree School, Catholic University of Valencia San Vicente Mártir, 46001 Valencia, Spain; joseluisplateroarmero@gmail.com; 3 Sandra Carrera-Juliá: Department of Nutrition and Dietetics, Catholic University of Valencia San Vicente Mártir, 46001 Valencia, Spain; sandra.carrera@ucv.es; 4 María Mar López-Rodríguez: Department of Nursing, Physiotherapy and Medicine, University of Almería, 04120 Almería, Spain; asta@um.es; 5 Interdisciplinary Laboratory of Clinical Analysis, Campus of Excellence Mare Nostrum, University of Murcia, 30100 Murcia, Spain; 6 Department of Nursing, Catholic University of Valencia San Vicente Mártir, 46001 Valencia, Spain; joseenrique.delarubi@ucv.es

**Keywords:** multiple sclerosis, feeding behaviour, anthropometry, PON1 human protein, interleukin 6

## Abstract

Background: Multiple sclerosis (MS) is a chronic neurodegenerative disease of an inflammatory, demyelinating and autoimmune nature. Diets with a high caloric density could be especially relevant in terms of the pathogenesis related to an increase in adipose tissue that is metabolically active and releases mediators, which can induce systemic inflammation and an increased oxidation state. The aim of this study was to analyse the eating habits related to calorie intake and their impact on abdominal obesity associated with anthropometric variables, the activity of the oxidation marker paraoxonase 1 (PON1), and interleukin 6 (IL-6) levelsin MS patients. Methods: An analytical and quantitative observational study was conducted with a population of 57 MS patients. The dietary-nutritional anamnesis was gained through the Food Frequency Questionnaire and a food diary. Diet and eating habits have been analysed through the Easy Diet–Programa de gestión de la consulta^®^ software. Anthropometric measurements were taken in order to determine the presence of abdominal obesity. In addition, PON1 was quantified in serum by means of automated spectrophotometric assays and IL-6 was quantified using the ELISA technique. Results: A normal calorie intake was determined for women, yet a slightly lower intake was observed in men. Carbohydrate consumption was below what was established, and protein and lipids were over, in both cases. Furthermore, most patients had abdominal obesity, with significantly higher body mass index (BMI), waist-to-hip ratio (WHR), waist-to-height ratio (WHtR), fat percentage and IL-6 levels. IL-6 is greatly correlated with waist circumference and WHtR. Conclusion: MS patients’ nutrient intake shows an imbalance between macronutrients. This seems to favour the abdominal obesity associated with high values of proinflammatory IL-6 that is not correlated with a lower activity of PON1.

## 1. Introduction

Multiple sclerosis (MS) is a neurodegenerative disease of an inflammatory nature and of an autoimmune cause that progresses with damage to the myelin that covers neurons [1]. Clinically speaking, MS patients show functional disabilities associated with muscle mass loss and an increase in fat mass [2].

The patients’ nutritional condition can affect the clinical development of the disease [3], especially functional disability as it is related to anthropometric parameters, such as body mass index (BMI) and waist circumference [4]. In this sense, abdominal fat accumulation is particularly important, as it has a direct contribution to the chronic inflammation state of the disease [5], mediated by the production of proinflammatory cytokines [6]. Amongst the cytokines related to MS, interleukin 6 (IL-6) takes the spotlight, due to its high levels in blood serum in MS patients [7] and the fact that it is related to the pathogenesis of the disease [8]. In addition, IL-6 is positively correlated with obesity [9], and especially with an increase in abdominal fat [10]. These metabolic alterations have an influence on oxidation state, modifying biochemical markers that are also related to inflammation, such as the enzyme paraoxonase 1 (PON1). Low levels of PON1 in serum are associated with the development of neurodegenerative diseases [11] and specifically with MS [12]. PON1 acts by inhibiting the oxidation of LDLs, preventing cytokine production from being triggered [13], which makes it an inflammatory marker with antioxidant properties [14] and proving to be especially efficient to assess the metabolic state [15].

In this sense, previous studies indicate that nutritional alterations occur among these patients [16]. Thus, it has been found that patients perceive how the severity of the daily manifestations of the symptoms of their disease is directly related to the excessive consumption of meat, fatty foods and processed sugars [17].Specifically, in pediatric MS patients, diets with high levels of fat increase the risk of [18].However, and despite the importance of diet for pathology, nutritional status and eating habits in MS patients have not been studied in depth, as of yet. Taking this into account, the aim of this study is to analyse the eating habits related to calorie intake and its impact on the abdominal obesity associated with anthropometric variables, PON1 activity and IL-6 levels in serum. In addition, we aim to determine the possible correlation of IL-6 levels with these anthropometric and biochemical parameters.

## 2. Materials and Methods

Across-sectional, analytical and quantitative observational study was conducted.

### 2.1. Subjects

In order to obtain the population sample, we contacted the main state-wide MS associations which informed their members on the nature of the study. The following selection criteria were applied to the 72 people interested in participating in the study: patients over 18 years of age diagnosed with MS at least 6 months ago and treated with glatiramer acetate and interferon beta. Moreover, the exclusion criteria included: pregnant or breastfeeding women, patients with tracheotomy, stoma or with short bowel syndrome, patients with dementia, evidence of alcohol or drug abuse, with myocardial infarction, heart failure, cardiac dysrhythmia, symptoms of angina or other heart conditions, patients with kidney conditions with creatinine levels two times higher than normal markers, patients with elevated liver markers three times higher than normal or with chronic liver disease, patients with chronic metabolic diseases, patients with acromegaly, patients with polycystic ovary syndrome or MS patients who were included in other researches with experimental drugs or treatment.

### 2.2. Statistical Analysis

A statistical analysis was performed with the SPSS v.23 (IBM Corporation, Armonk, NY, USA) tool. The first step had the objective of estimating the distribution of the variables investigated through statistical methods in order to assess normality, including the Kolmogorov—Smirnov Test. This analysis demonstrated the non-normal distribution of all the scale variables that had been studied. Therefore, the chi-square test and the Mann–Whitney U test were used to analyse categorical and numerical data, respectively. Finally, the Spearman test was used to find a possible correlation between the variables. A *p*-value below 0.05 was considered to be significant. Data are presented as mean ± standard deviation, or the number of patients and percentage.

### 2.3. Procedure

Once the sample was obtained, the volunteers and their families received detailed information on the study. The participants then signed an informed consent form after accepting to take part in the study. 

### 2.4. Measurements

The following measurements were taken throughout the study:

#### 2.4.1. Dietary-Nutritional Anamnesis

The Food Frequency Questionnaire was used [19], which gave us information on how often different food groups were consumed: dairy products, vegetables, fruit, juices, nuts, meat, fish, seafood, eggs, tubers, rice, legumes, pasta, cold meats and sausages, snacks, pastries and biscuits, chocolate bars, soft drinks, fermented alcohol and distilled alcohol. The self-administered questionnaire asked about how oftencertain food groups were usually consumed in a week.

In addition to using a food diary, each patient registered their solid and liquid food intake for 7 days. This period of time enabled us to gather sufficient information on the patients’ normal diet, minimising the risk of bias associated with choosing one day a week [20]. Patients wrote down the type of food they consumed, as well as all the different ingredients used to make each and every dish. Each participant also took note of the amount that was consumed per intake, indicating the household measurement (a cup, a portion, a glass, a tablespoon, a slice, a handful, a plate, a ladleful…) or the exact weight of the food or drink. In order to make it easier for them to complete the task, patients were provided with information regarding the weight for each portion and the most common household measurements [21].

#### 2.4.2. Diet and Eating Habit Analysis

Taking into account the patients’ food diary over 7 days and the Food Frequency Questionnaire, the quality of the diet was calibrated by using the Easy Diet–Programa de gestión de la consulta^®^ software. With this software, a nutrition profile containing the daily averageof energy intake, total proteins, carbohydrates, carbohydrate profile (simple carbohydrates), total lipids, lipid profile (monounsaturated, saturated and polyunsaturated fatty acids), cholesterol and percentage distribution of macronutrients (proteins, lipids and carbohydrates)was obtained from the meals introduced to the program by the nutritionists. Additionally, in order to assess whether the diet was adequate or not, DRI (Dietary Reference Intake) from “Ingestas de referencia para la población Española” [22] and “Consenso de la Sociedad Española de NutriciónComunitaria (SENC)” [23] were taken as guidelines.

#### 2.4.3. Identification of Abdominal Obesity and Anthropometric Variables

Body weight, height, waist circumference and hip circumference were measured following the protocol established by The International Society for the Advancement of Kinanthropometry (ISAK) [24] by an ISAK level 3 certified anthropometrist. A portable clinical scale SECA model, with a 150–200 kg capacity and 100 g precision was used, as well as a stadiometer, model SECA 220 Hamburg, Germany, with a 0.1 cm precision and a metal, inextensible and narrow anthropometric tape, model Lufkin W606PM with 0.2 mm precision [25].

The following were calculated with the obtained data: BMI [26], waist-to-hip ratio (WHR) [27] and waist-to-height ratio (WHtR). Abdominal obesity was identified based on WHtR when it was over 0.5 [28].

#### 2.4.4. Biochemical Analysis

Blood samples were taken from the antecubital vein at 11 a.m. on an empty stomach and were collected in BD Vacutainer Plus serum blood collection tubes (ref. 367815). The samples were then kept at room temperature for 30 min in order to coagulate. The coagulated part was then separated by centrifuging the samples at 4000 rounds/min for 10 min in a refrigerated centrifuge. After centrifuging, the supernatant liquid was transferred to 0.5 mL aliquots that were frozen and stored at −80 °C. Finally, once the samples thawed, the concentration of IL-6 in serum was determined by means of the ELISA technique (R&D Systems). PON1 activity was determined by using 4-nitrophenyl acetate with an automatic clinical biochemistry analyser (Olympus A 400, Tokyo, Japan) [29].

#### 2.4.5. Expanded Disability Status Scale (EDSS)

This scale is used to assess functional disability in MS patients [30]. It is an ordinal scale based on a neurological examination of the eight functional systems (pyramidal, cerebellar, brainstem, mental, sensory, visual, bowel and bladder), together with assessing walking capacity, which provides a disability index between 0 and 10 as a result. In terms of calculation, 0 is understood as having normal health and 10 is understood as death by MS.

### 2.5. Ethical Concerns

The study was developed in accordance with the Declaration of Helsinki [31], with the prior approval of the protocol by the Human Research Committee of the University of Valencia of the Experimental Research Ethics Committee (procedure number H1512345043343). Participants were provided with a written informed consent form after being informed of the procedures and the nature of the study.

## 3. Results

After applying the selection criteria indicated in the Material and Methods section, and once some patients had abandoned the intervention, this study analysed a sample of 57 MS patients with an average age of 47.04 years, of which 66.7% were women and 33.3% were men. Thirty-seven patients had relapsing-remitting MS and 14 had secondary progressive MS, and with a functional capacity of 3.86±2.0 in the EDSS test. 

Biochemical markers (IL-6 and PON1) and anthropometric variables (weight, height, BMI, waist circumference, hip circumference, WHR and WHtR) are shown in Table 1 below. 

### 3.1. Description of Eating Habits 

In accordance with the data obtained and once the daily diet and eating habits of the population of the study were analysed, the mean calorie intake for women was 1917.11 kcal and 2312.04 kcal for men. 

When analysing the percentage distribution of ingested macronutrients regarding the total calorie volume, with regards to lipids, 42.14% was observed in women and 41.96% in men, of which the mean intake of monounsaturated fatty acids was 34.25% for women and 37.56% for men, polyunsaturated fatty acids was 26.77% for women and 25.52% for men, and saturated fatty acids was 29.42% for women and 28.55% for men. Finally, the mean for cholesterol in women was 299.27 mg and 356.88 mg for men (Table 2). In terms of carbohydrate intake, it was 38.61% for women and 40.04% for men, with a mean percentage of simple carbohydrates intake of 50.66% for women and 46.94% for men (Table 2). Regarding proteins, women had an intake of 23.81%, while men ingested 29.79% of the total calorie volume (Table 2). 

### 3.2. Abdominal Obesity: Anthropometric and Biochemical Values 

As observed in Table 3, 50 patients (87.7%) were considered to have abdominal obesity, while 7 (12.3%) were not classified as having it.

The average levels of IL-6 are significantly higher when patients have abdominal obesity. There are no differences, however, in PON1 values between both groups of patients. BMI, body fat percentage, waist circumference, WHR and WHtR were substantially higher in patients with abdominal obesity, in comparison to those who did not. However, there were no differences in hip circumference. Finally, no important differences were observed between both groups in terms of age, weight and height.

### 3.3. Correlation between the Levels of IL-6, with the Analysed Anthropometric Variables and PON1

Regarding the correlations of IL-6 values as an inflammation marker with the analysed parameters, a positive correlation has been observed with waist circumference and WHtR. Therefore, the higher the levels of interleukin in serum, the higher the values of these variables. On the other hand, there is no significant correlation between BMI, WHR, hip circumference, fat percentage and PON1 (Table 4). 

## 4. Discussion

Macronutrient intake can help or hinder the progression of diseases of a demyelinating nature such as MS [32], as it determines patients’ nutritional condition related to the pathology of the disease [3]. Due to this, our study analysed the calorie intake of the participants’ diet, and determined that women’s mean calorie intake is within the recommended dietary reference intake (DRI) (1875–2300 kcal). However, the mean calorie intake for men was slightly lower than the recommended minimum (2400–2850 kcal) [22]. Nonetheless, percentage distribution in macronutrients (proteins, carbohydrates and lipids) did not meet the criteria of a balanced diet for both men and women [23], alongside a high disability level established by the EDSS test. In terms of carbohydrates, both sexes showed an intake lower than the recommended minimum (50%–55%) regarding the total calorie volume, yet simple carbohydrate intake was clearly higher than recommended (5%–10%) [23]. Nevertheless, the mean protein percentage for both men and women exceeds DRI recommendations (10%–20%) and the mean lipid percentage also surpasses the recommended values (30%–35%) [23]. In addition, the lipid profile for both sexes is characterised by an intake of MUFA, PUFA and SFA that does not comply with the DRI [22], alongside a higher consumption of cholesterol in men, which is in line with other publications by other authors [33]. In this sense, certain studies have observed that there is a link between the dietary intake of lipids and a higher prevalence and progression of MS [34,35]. These eating habits (and derived anthropometric characteristics) may be related to variables associated with the disease itself. These include treatment, mainly based on the administration of immunomodulators, and glucocorticoids that produce changes in food intake and weight gain [36], based on an increase in the percentage of abdominal fat [37]. In addition, the role of interleukin IL-6 itself, linked to the pathogenesis of the disease, has an important role in energy homeostasis [38] and whose acute central administration reduces intake in the short term, while when it is chronic central administration, it induces a reduction in intake and a loss in fat mass [39].

An unbalanced diet alongside other environmental variables and factors, especially physical activity, could contribute to the anthropometric characteristics of the study population. Therefore, abdominal obesity was observed in a high number of patients, in which adipose tissue in the abdomen was associated with a significantly higher BMI, waist circumference, WHR, WHtR and fat percentage, in comparison to patients with no abdominal obesity. Similar results were registered in the study by Susan K. et al. 2017, in which BMI was positively associated with protein and lipid intake and negatively with carbohydrate intake in obese patients [40]. In addition, excessive simple carbohydrate intake is related to obesity and being overweight [41], as well as an increase in adipose tissue in the abdomen [42]. 

In terms of the impact and influence of dietary intakes on the level of inflammation, we must highlight the fact that an excessive intake of lipids is related to oxidative stress and inflammation [43,44]. In addition, at a clinical level, one of the main symptoms of the disease is the motor-level alterations related to anthropometric alterations observed in the disease [45]. In this sense, an adequate intake of lipids has been observed to improve the feeling of fatigue in MS patients [46].Therefore, among the practical implications of this study, we found that establishing new dietary guidelines based on decreasing high lipid intakes could be a therapeutic strategy to reduce the inflammatory state associated with fatigue, involving a possible motor improvement.

This decrease in lipid levels should also be accompanied by a lower percentage of simple carbohydrates that are also directly related to the production of proinflammatory cytokines [47]. Amongst these cytokines, our study determined it was IL-6 whose levels were high. These results coincide with those published by other authors [7]. Regarding the relation to anthropometric variables, there is a positive correlation with WHtR and waist circumference, while this did not happen with fat percentage, WHR, hip circumference and BMI. It seems to indicate that IL-6 is positively associated with fat accumulated in the abdominal area, as observed by other authors [10]. This is verified with significantly higher levels of interleukin in patients with this kind of obesity, which is also linked to the inflammatory status of the disease [5]. In this sense, the fundamental role of IL-6 in the inflammatory process of the pathology seems to be confirmed, which is in line with other results that links it to the pathogenesis of MS [8].

However, we must highlight that, in our study, this increase in IL-6 is not correlated with PON1 levels. This result indicates that the accumulation of fat, particularly in the abdomen area, is related to higher values of IL-6. However, it is not related to PON1 activity as a marker of total oxidation state. Thus, despite PON1 being a marker sensitive to anthropometric and metabolic improvements after nutritional treatment in MS patients, [48] and that its antioxidant activity is low in the disease [12], it is not a good marker for oxidative stress associated to inflammation for this disease, coinciding with what has been observed by other authors [49].

## 5. Conclusions

Regarding nutrient intake in MS patients, despite calorie intake being within (women) or slightly lower (men) than the recommended values, there is an imbalance with carbohydrate intake being lower and lipid and protein intake being higher than the established recommendations. This seems to favour abdominal obesity associated to high values of proinflammatory interleukin 6, which, however, is not correlated with a lower activity of the oxidation marker PON1.

Despite these results, we have found that our study has its limitations, which include the fact that the number of patients was not very high and only MS patients from Spain have been considered. In this sense, we believe that it would be a good idea to conduct a study that covers patients from different countries, allowing to also compare and analyse the impact of different food characteristics from other nationalities and regions. In addition, we think that other markers should be analysed, both for inflammation and oxidation, in order to establish the possible adequacy when determining the influence of the anthropometric characteristics of this population.

## Figures and Tables

**Table 1:** Biochemical and anthropometric values analysed in the population sample.

	Women		Men		Total	
	Mean	SD	Mean	SD	Mean	SD
IL-6 (pg/mL)	3.44	3.07	3.96	4.21	3.43	3.27
PON1(UI/L)	2.80	0.70	3.06	0.60	2.86	0.68
Weight (kg)	67.37	13.91	75.59	16.28	69.94	15.04
Height (cm)	162.00	6.60	171.84	6.35	165.26	7.91
BMI (kgm^2^)	25.80	5.10	25.69	5.92	25.70	5.31
Waist circumference (cm)	96.18	13.13	94.49	10.37	95.40	11.81
Hip circumference (cm)	108.70	12.45	98.81	10.69	104.14	12.56
WHR	0.89	0.09	0.96	0.08	0.92	0.09
WHtR	0.60	0.08	0.55	0.07	0.58	0.08

Il-6: Interleukin 6 (mean value of normal IL-6 1.4 pg/mL); PON1: Paraoxonase 1; BMI: Body Mass Index; WHR: waist-to-hip ratio; WHtR: waist-to-height ratio; SD: Standard Deviation.

**Table 2:** Description of eating habits and daily calorie intake of the population of the study.

	Women Study		DRI Women	Men Study		DRI Men
	Mean	SD		Mean	SD	
Energy (kcal)	1917.11	399.74	1875–2300	2312.04	468.91	2400–2850
Proteins (g)	91.94	20.70	46	94.16	17.68	56
Proteins (%)	23.81	6.70	10–20	29.79	7.44	10–20
Lipids (%)	42.14	5.52	30–35	41.96	3.31	30–35
MUFAs (%)	34.25	3.60	20	37.56	4.15	20
PUFAs (%)	26.77	5.12	5	25.52	5.35	5
SFA (%)	29.42	6.29	7–8	28.55	5.05	7–8
Cholesterol (mg)	299.27	82.80	<300	356.88	129.10	<300
Carbohydrates (g)	182.83	38.40	130	227.87	61.18	130
Carbohydrates (%)	38.61	5.15	50–55	40.04	4.10	50–55
Simple carbohydrates (%)	50.66	13.06	5–10	46.94	10.51	5–10

DRI: dietary reference intake; MUFAs: monounsaturated fatty acids; PUFAs: polyunsaturated fatty acids; SFA; saturated fatty acids; SD: Standard Deviation. The percentage of the different macronutrients is expressed in reference with the total calorie volume. For simple carbohydrates, the percentage is expressed in reference with the total carbohydrate intake.

**Table 3:** Comparison between patients with or without abdominal obesity, according to waist-to-height ratio (WHtR).

	Without Abdominal Obesity *n* = 7		With Abdominal Obesity *n* = 50			
	Mean	SD	Mean	SD	Z	*p*
Age (years)	41.00	18.46	51.48	9.36	−1.547	0.122
IL-6 (pg/mL)	1.77	0.80	3.79	3.43	−2.087	0.037 *
PON1 (UI/L)	2.97	0.20	2.90	0.71	−0.386	0.700
Weight (kg)	67.86	7.85	77.66	14.34	−1.555	0.120
Height (cm)	172.80	4.49	165.47	9.17	−1.661	0.097
BMI (kg/m^2^)	22.87	3.49	28.54	5.02	−2.394	0.017 *
Waist circumference (cm)	80.90	5.27	97.53	10.99	−3.215	0.001 *
Hip circumference (cm)	100.00	8.60	104.74	13.02	−0.673	0.501
WHR	0.82	0.12	0.94	0.07	−2.338	0.019 *
WHtR	0.47	0.02	0.59	0.07	−3.576	0.000 *
Fat percentage	16.68	3.00	21.36	4.30	−2.052	0.040 *

IL-6: Interleukin 6; PON1: Paraoxonase 1; BMI: Body Mass Index; WHR: waist-to-hip ratio; WHtR: waist-to-height ratio; Z: Mann–Whitney U test; * statistically significant differences *p* < 0.05.

**Table 4:** Correlations of Interleukin 6 (IL-6) with anthropometric variables and paraoxonase 1 (PON1).

		PON1	BMI	Waist Circumference	Hip Circumference	WHR	WHtR	Fat Percentage
IL-6	Coef.	0.318	0.217	0.407	0.194	0.291	0.413	0.235
	*p*	0.058	0.152	0.028 *	0.313	0.125	0.026 *	0.135

IL-6: Interleukin 6; PON1: Paraoxonase 1; BMI: Body Mass Index; WHR: waist-to-hip ratio; WHtR: waist-to-height ratio; Coef.: Spearman’s correlation coefficient; * statistically significant correlations *p* < 0.05.
